# A Discrete Event Simulation Model for Evaluating the Performances of an *M/G/C/C* State Dependent Queuing System

**DOI:** 10.1371/journal.pone.0058402

**Published:** 2013-04-01

**Authors:** Ruzelan Khalid, Mohd Kamal M. Nawawi, Luthful A. Kawsar, Noraida A. Ghani, Anton A. Kamil, Adli Mustafa

**Affiliations:** 1 School of Quantitative Sciences, Universiti Utara Malaysia, Sintok, Kedah; 2 School of Distance Education, Universiti Sains Malaysia, Penang, Malaysia; 3 School of Mathematical Sciences, Universiti Sains Malaysia, Penang, Malaysia; 4 Department of Statistics, School of Physical Sciences, Shahjalal University of Science and Technology, Sylhet, Bangladesh; Cinvestav-Merida, Mexico

## Abstract

*M/G/C/C* state dependent queuing networks consider service rates as a function of the number of residing entities (e.g., pedestrians, vehicles, and products). However, modeling such dynamic rates is not supported in modern Discrete Simulation System (DES) software. We designed an approach to cater this limitation and used it to construct the *M/G/C/C* state-dependent queuing model in Arena software. Using the model, we have evaluated and analyzed the impacts of various arrival rates to the throughput, the blocking probability, the expected service time and the expected number of entities in a complex network topology. Results indicated that there is a range of arrival rates for each network where the simulation results fluctuate drastically across replications and this causes the simulation results and analytical results exhibit discrepancies. Detail results that show how tally the simulation results and the analytical results in both abstract and graphical forms and some scientific justifications for these have been documented and discussed.

## Introduction

*M/G/C/C* state dependent networks are typical systems in our life. The examples include pedestrians flow through corridors, vehicles movement on roads, products delivery through accumulating conveyers, etc. Here the term M/G/C/C state dependent means that inter-arrival time distribution is Markovian, service time follows a General distribution, which is dependent on the number of customers in the system, C parallel servers and a total capacity of C. Since the service time depends on the number of residing entities (i.e., pedestrians, vehicles, products, etc.), we can control the system's service time and throughput through their arrival rates. Slow arrival rates cause less residing entities and thus make them to be serviced faster. This however causes little throughputs at certain period of time. High arrival rates increase the number of residing entities and thus make them to be serviced slower. This however may increase the throughputs at the end. A higher value of arrival rates than its capacity tolerance limit will cause congestion. This situation tends to create havoc instead of improving the throughput. Thus, controlling the arrival rates so that the throughput of the system is optimized is crucial especially in an emergency evacuation case.

Yuhaski and Smith have presented linear and exponential models for uni-directional service times in terms of walking speed as follows [1]:

Linear: 
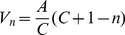
(1)


Exponential: 
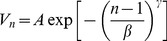
(2)


where 
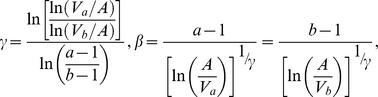



*γ, β* =  Shape and scale parameters for the exponential model,

*V_n_* =  Average walking speed for n pedestrians in a corridor,

*V_a_* =  Average walking speed when crowd density is 2 ped/m^2^ = 0.64 m/sec,

*V_b_* =  Average walking speed when crowd density is 4 ped/m^2^ = 0.25 m/sec,

*A = V_1_* =  Average walking speed when there is a single pedestrian = 1.5 m/sec,

*n* = Number of pedestrians in a corridor,

*a* = *2* × *L* × *W*,

*b* = *4* × *L* × *W*,

*C* = *5* × *L* × *W*,

*L* =  Length of the corridor, and

*W* =  Width of the corridor.

Based on the models, Cheah [2] developed the limiting probabilities for the number of pedestrians in an *M/G/C/C* state dependent queuing model as follows:
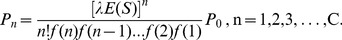
(3)


where, 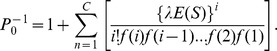


In this model, *E(S)* is the expected service time of a single pedestrian in a corridor of length *L*, *P_n_* is the probability when there are *n* pedestrians in the corridor, *P_0_* is the probability when there is no pedestrian in the corridor, and *f(n)* is the service rate and is given by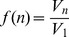. C meanwhile refers to the capacity of the corridor. Tregenza [3] showed that the capacity is equal to the highest integer that is less than five times the area of the corridor in square meters. Any pedestrians attempting to enter a full capacity corridor will be blocked. The probability of such blocking (*P_balk_*) is equal to *P_n_* where *n* equals *C*. The different performance measures of the corridor can then be computed as
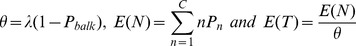



where 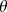 is the steady state throughput through corridor, *E(N)* is the expected number of pedestrians in the system and *E(T)* is the expected service time in seconds.

Kawsar *et al.* [4] used an *M/G/C/C* model to evaluate performances of pedestrian traffic flow within a complex topological network that is the Dewan Tuanku Syed Putra (DTSP) hall room of Universiti Sains Malaysia. Their main premise is that its throughput can be increased by controlling the arrival rate to each of its source corridors and such control is crucial in an emergency case, e.g. fire, explosion, etc. Based on the optimal arrival rate, performances of each source corridor and its relevant exit corridors in terms of their throughputs, blocking probabilities, expected service time and expected number of entities have been documented and discussed in details.

Analytical results of the network can be validated using a discrete event simulation model [5], [6]. In this model, pedestrians (entities) seize a unit (a space in a corridor) of available servers (the capacity of the corridor) and delay it as a function of the current number of busy servers (the number of residing pedestrians). The unit will be released once the pedestrian seizing it has finished its travel time and later be seized by another pedestrian. This kind of mechanism can flexibly be programmed using any procedural or object oriented programming (OOP) languages, e.g. C [7], Java [8], C++ [9], [10], etc. and has been focused and discussed in detail in the previous paper [11]. However, constructing basic libraries for structuring and running the model (e.g. simulation clock, simulation calendar and engine, distribution types, statistical reports, etc.) and embedding animations for getting insight into its inner processes (that is to show the pedestrians' behavior and flows over time) will demand programming experiences and consume time. Modern simulation software offers libraries and facilities for the model's structures, animation and analysis either in abstract and graphical forms. However, their inner workings are only based on common queuing mechanisms, that is servers' service time cannot be changed once they have been seized by entities.

Most simulation software only permits us to specify entities' service times or servers' processing times based on certain distributions, e.g. Exponential, Poisson, Gamma, etc. The service times determine how long they will seize (be delayed by) the servers and any updates during these times are not allowed. Such mechanism limits us from representing the *M/G/C/C* networks that consider the entities must dynamically be delayed as a function of the number of seized servers. Thus, the main contribution of this paper is the approach how to support this important feature using most simulation tools. Other contributions include the thorough investigations and reports on the range that the simulation and analytical results will exhibit some discrepancies.

We organized this paper as follows. The subsequent section briefly discusses the main limitation of commercial simulation tools in modeling *M/G/C/C* networks and presents ideas how this limitation can be tackled. Further, we focus on the modeling of the networks using modules available in Arena software. In the following section the simulation results are compared with analytical results of the selected complex topological network. Reports on how tally the simulation results and the analytical results in abstract and graphical forms and some discussion on this are documented and discussed. Finally, the last section summarizes the findings and presents some conclusions.

## Materials and Methods

### Discrete Event Simulation Model

Any simulation software can be tailored to model the *M/G/C/C* networks. However, we have not found any report on how this is possible. Our main challenge is to dynamically update the pedestrians' service rates as a function of their density in a corridor, since most simulation tools do not permit the increment or decrement of a server's delay time (processing time) once it has been in a busy state. Alternatively, we could model the networks using a conveyer approach where its length and velocity are based on the capacity of the corridor and the number of pedestrians residing in it. However, since the velocity cannot also be changed during run time, this limits us from further investigation on how these conversions are possible. In spite of this fact, there has been some researches utilizing the *M/G/C/C* mathematical model with its states is set constant to evaluate the performances of material handling systems in which accumulating conveyers are used to deliver products (e.g. see [12], [13]).

To solve this, we can store pedestrians in a queue. The waiting time they spent in the queue represents their travel time through a corridor and its buffer size (that is the maximum number of pedestrians that can enter the queue) represents the capacity of the corridor. Full capacity blocks pedestrians from entering the queue and accumulates them in another queue. Whenever there is an event in the queue (that is entrance and departure of a pedestrian), two things will happen. First, the current walking speed of the corridor, *V_n_* needs to be updated and the value must be assigned to all other residing pedestrians. Second, the pedestrians have to calculate their *delay time* (remaining time) to exit the corridor that is by considering their remaining distance to cross the corridor and the current value of the *V_n_*. Thus, pedestrians should have attributes as listed in Table 1. Note that the formula is used to update a pedestrian's current location in a corridor based on the previous walking speed whenever a new pedestrian enters or an existing pedestrian leaves the corridor.

**Table 1:** Pedestrians' Attributes.

lastLocation = lastLocation + V_n−1_ × (currentEventTime – lastEventTime)
lastEventTime = currentEventTime
delayTime = (lengthOfCorridor − lastLocation)/V_n_
occurTime = currentEventTime + delayTime

To implement this logic, a simulation tool should support a mechanism for removing entities from their queue so that their states can be updated and a mechanism for delaying their delay times so that they can be freed whenever their occurred times have been reached.

In addition to these attributes, pedestrians should also have other auxiliary attributes that measure the time that they have spent in the corridor that is *timeEnterCorridor*, *timeExitCorridor*, *timeSpentInCorridor*, etc. The *timeEnterCorridor* stores the time a relevant pedestrian enters the corridor, *timeExitCorridor* stores the time he/she exits the corridor while the *timeSpentInCorridor* stores the time that he/she has spent to cross the corridor (that is *timeExitCorridor – timeEnterCorridor*). Using this logic, the performances of the corridor can be stored in relevant variables and evaluated using the relationships listed in Table 2.

**Table 2:** Model's Variables.

*p(c)* = sumBlockedPedestrians/sumArrivalPedestrians
θ = sumDepartedPedestrians/simulationLength
*L* = sumTimeSpentInCorridybyAllPedestrians/simulationLength
*W* = sumTimeSpentInCorridybyAllPedestrians/sumDeparture

### Arena as an Implementation Tool

**Figure 1:**
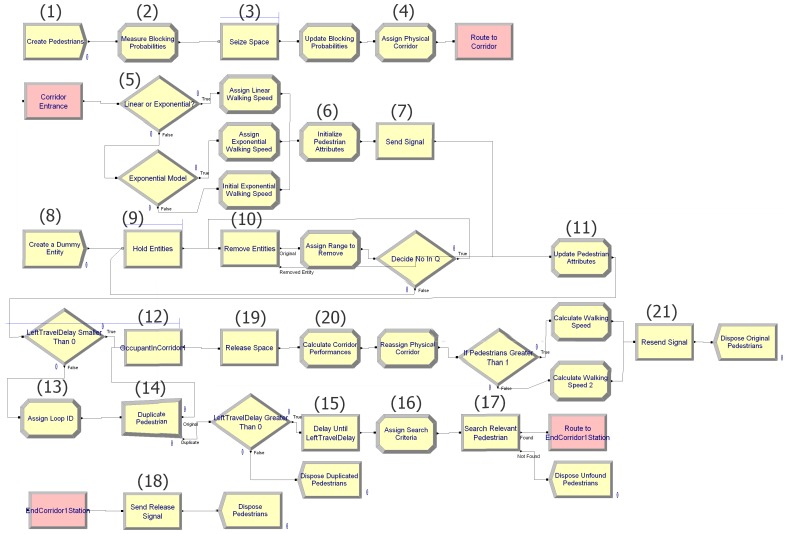
Arena model for *M/G/C/C* networks.

We used Arena [14], [15], [16], SIMAN-based simulation software, to model the *M/G/C/C* networks. Besides the fact that the software does not allow us to variably change a server's service time once it has been seized by an entity, it also offers no direct access to entities in queue. Fortunately, it provides a module for removing entities from their queue. This feature enables us to remove, update their current states (e.g. their current locations, delay time, etc.) and flow them back to the queue. Figure 1 shows our basic model for the *M/G/C/C* networks. Although it is implemented in Arena, the structures and logic of flowing pedestrians throughout their lifecycles are straight forward and can easily be implemented in any other DES software, e.g. SIMUL8 [17], ExtendSim [18], etc.

We first create a sample of pedestrians according to the exponential distribution using the *Create* module (1). Their creations are based on time between arrivals. Thus, we have to convert the λ (that is pedestrian arrival rate in the mathematical models) to 1/λ in order to present the time between their arrivals to the corridor. This 1/λ should also be specified as the model's first event that is the creation time for the first pedestrian. However, omitting this will not affect the model's performances since it will be run for a long period of time (that is until its steady state). Besides these pedestrians, we have to create one dummy entity (the *Create* module (8)) that will iteratively activate a mechanism to remove pedestrians and update their current states.

Every arrival must be counted. For this, we used an *Assign* module. It allows us to declare and assign relevant values to variables or attributes, and has been used throughout the model to update its states. In the *Assign* module (2), we defined variables that respectively store the number of pedestrians that have entered and that have been blocked from entering the corridor for calculating its blocking probability (see Table 2), and an attribute that stores their identification numbers (IDs) for later use in the model. The IDs are assigned based on the number of pedestrians in the system, and this global variable must always be updated every time a new pedestrian arrives at the system.

Each pedestrian tries to seize a space in the corridor. This situation can be presented using the *Seize* module (3) that allocates a unit (a space) of available servers (the capacity of the corridor) to the pedestrian. The capacity of the corridor (that is 5× length × width) has first to be declared in another place (e.g. using the *Expression* spreadsheet). If all available units are busy, the pedestrian will automatically be queued until the unit is available to be seized.

Every successful pedestrian will initially be introduced to the physical corridor. The *Assign* module (4) defines variables relating to this, that is its area, its capacity and its current number of residing pedestrians and will be used to calculate the current travel speed in the corridor. Since there are two mathematical models for calculating the speed that is linear and exponential models (see Equation (1) and Equation (2)), we used the *Decide* module (5) to offer the option. However, only the exponential models were used to analyze and report the analytical and simulation results in this paper. In the *Exponential Model* decision module, we have to use the *Initial Exponential Walking Speed* and *Assign Exponential Walking Speed* blocks to clearly differentiate the speed for a single pedestrian (that is 1.5m/s) and the speed for occupied pedestrians that are greater than one in a relevant corridor. We have to do this since Arena software only has a built-in mathematical function for an exponential function with base *e*. Thus, we have to convert 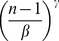
in the Equation (1) to 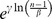. However, this natural logarithmic is undefined if 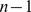 = 0, that is when there is a single pedestrian in a corridor.

When pedestrians start travelling through the corridor, we have to initialize their entrance time to the current simulation clock value and their current travel distance to zero. This is accomplished by the *Assign* module (6). Simultaneously, a signal (that denotes the arrival event) needs to be sent using the *Signal* module (7) to force the *Hold* module (9) (that is a type of queue that releases its residing entities when receiving a signal or satisfying a condition) to release the dummy entity and then activate the *Remove* module (10). The *Remove* module removes pedestrians from their queue (the *Queue* module (12)) in order to update (the *Assign* module (11)) their states that is their current travel distance, their remaining time to exit the corridor and the time points that these events happen. This time will later be used for calculating the pedestrians' new states (see Table 1). We also need to assign (the Assign module (13)) their current number of state changes (loop IDs) that will be used as a search criterion later in the model. After performing the task, the dummy entity flows back to the *Hold* module (9) and waits for another signal.

The pedestrians cannot perform their delay times (remaining times to exit the corridor) while in queue. To solve this, we can duplicate them using the *Separate* module (14). The original pedestrians flow back to their queue after updating their states, while their clones (that have the same attributes and values) perform their delay time using the *Delay* module (15). After the delay time, they enter the *Assign* module (16) where the values of their IDs and loop IDs are assigned to new variables and used as search criteria (accomplished by the *Search* module (17)) to match their original pedestrians that satisfy both values.

The result of the search is either true (found) or false (not found). If the original pedestrian was not found, the duplicated pedestrian will instantly be destroyed to claim computer memory spaces. Else, it will send a signal (that denotes the departure event) using the *Signal* module (18) to the *Hold* module (9). The *Hold* module then releases the satisfying pedestrian from its queue that then frees (the *Release* module (19)) his/her space to be seized by other pedestrians. Before being destroyed, the pedestrian measures the performance of the corridor using the *Assign* module (20) and sends a signal (the *Signal* module (21)) to the *Hold* module (9) to force all pedestrians to update their new states.

We cannot control the cross lines from the *Remove* module (10) to the *Assign* module (11) since the *Remove* module has two exit points, that is the *Original* exit point to route the dummy entity to a decision block to wait for the next events or to iteratively remove pedestrians from their queue, and the *Removed Entity* exit point to route the removed pedestrians to update their states and return to their queue.

The basic model can easily be extended to support *series*, *splitting* and *merging* topologies. Since these topologies relate to the flow of pedestrians through various corridors, we should provide relevant mechanisms to perform these logics. First, we have to create a unique queue for each corridor so that we can store its residing pedestrians and update them accordingly, e.g. when there is an arrival or a departure event. Second, we have to attach an attribute to the pedestrians (e.g. *toCorridor* that will take their next corridor number) so that we can travel them correctly from corridor to corridor. The value of the attribute must be updated once a relevant pedestrian exits its current corridor and used throughout the model to support the logical statements of the model, e.g. when we want to remove or search pedestrians in their queue. Third, we have to create and send a unique signal number every time the pedestrian enters/exits their corridor to enable us to update their current states in the corridor.

**Figure 2:**
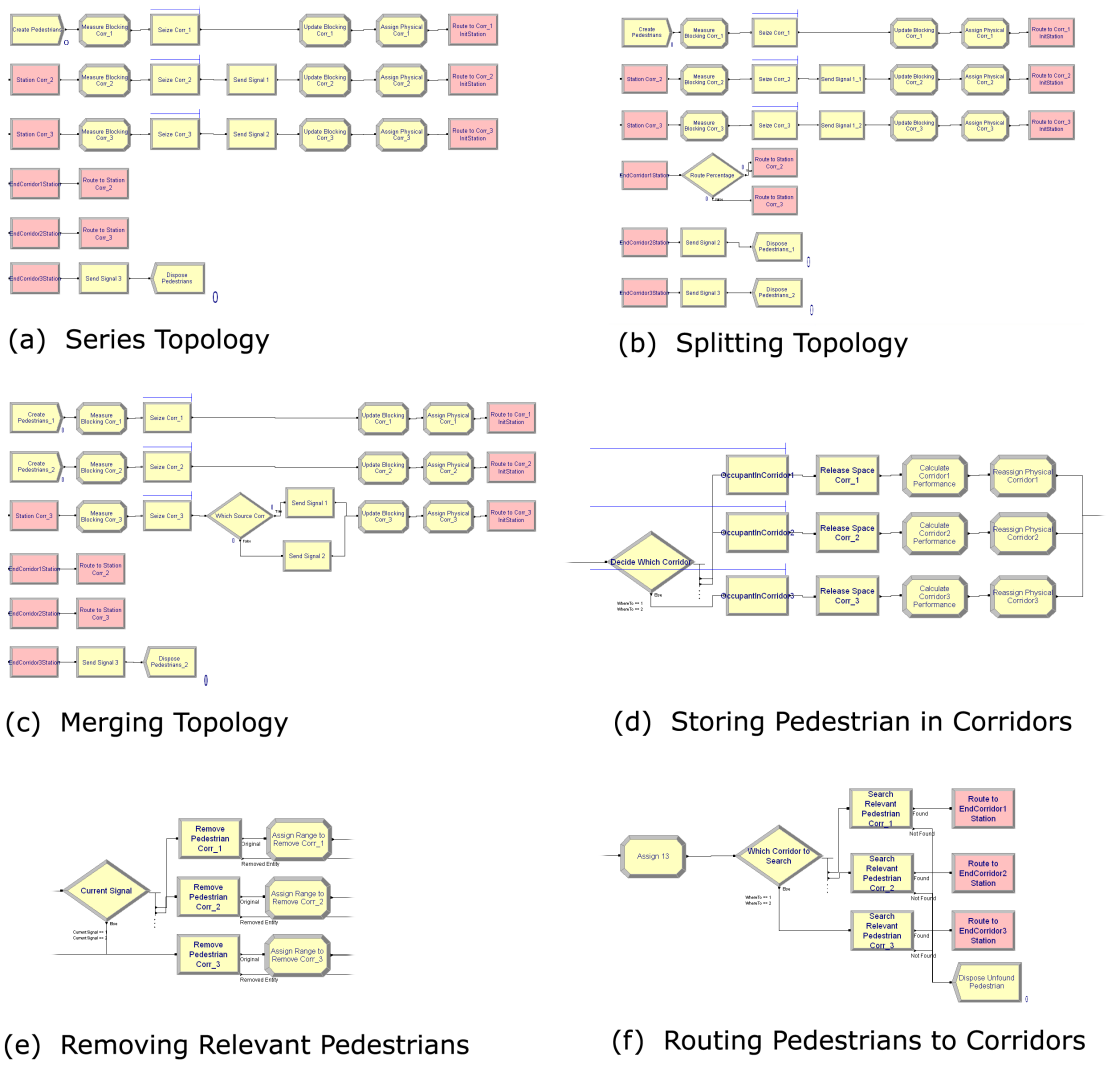
Structures of Series, Splitting and Merging Topologies in Arena. (a) Series Topology. (b) Splitting Topology. (c) Merging Topology. (d) Storing Pedestrian at a Relevant Corridor. (e) Removing Relevant Pedestrian. (f) Routing Pedestrian to Relevant Corridor.

Figure 2 shows snapshots of the three topology structures. Figure 2 (a to c) show Arena modules used for flowing pedestrians from corridor to corridor in series, splitting and merging corridors respectively. The structures are straightforward and can easily be comprehended by model designers who are familiar with high level DES software. Figure 2 (d to f) meanwhile show the modifications that must be made to our previous engine in order to store pedestrians in, remove pedestrian from and route pedestrians to relevant corridors. All the three topologies share the same engine.

## Results and Discussion

### Comparison of Analytical and Simulation Results

We run our simulation model using *Process Analyzer*. This application eases the analysis and comparisons of simulation results based on different model inputs. Our model's input controls are arrival rate, the length of a corridor, its length and width and pedestrians' average travelling distance since we have many source inputs of a corridor. The output responses are its blocking probability, throughput, expected number of pedestrians and their mean travel time.

All simulation results documented in this paper were carried out for 20000 seconds, and 10 and 30 replications respectively. We purposely ran each scenario for two different replication numbers to investigate if there would be any improvement of its outputs' half widths. Before being used to validate the analytical results of the considered networks [4], we first compared our simulation results with the simulation results reported in the previous paper [11]. We found that our simulation results were only less than 5% difference with theirs. Our simulation model also reported almost the same results as theirs for the obvious discrepancies results between analytical and simulation models (when λ = 5 pedestrian/second, length  = 8 meters and width  = 4.5 meters) that we noticed in the paper as in Table 3.

**Table 3:** Comparison of Analytical and Two Simulation Results.

Model	*p(c)*	*θ*	*L*	*W*	*CPU (s)*
Analytic	0.11	4.45	95.66	21.49	-
Simulation (Cruz et al., 2005) 95% CI	0.00 [0.00, 0.00]	4.99 [4.97, 5.00]	46.80 [45.54, 48.05]	9.39 [9.10, 9.68]	1100
Simulation (our) 95% CI	0.01 [0.00, 0.02]	4.96 [4.91, 5.02]	48.23 [43.71, 52.75]	9.80 [8.62, 10.98]	5142

Table 3 also reports the CPU (Central Processing Unit) time consumed by both models for running the scenario. We can observe that Cruz's model ran faster (1100 seconds using CPU Pentium II 400 MHz, 64 MB RAM, under Windows NT 4.00) than our model (5142 seconds using CPU Intel 2 Core Duo, 2.00 GHz, 2GB RAM, under Windows XP Professional). Other analyses on CPU times showed that their model is better than our model (in terms of speed) for any scenario that its arrival rates create blocking. We expected this since our model structures involve storing, searching and removing pedestrians from a queue while their model can directly access pedestrians in a queue to update their states and has implemented an optimization technique.

**Figure 3:**
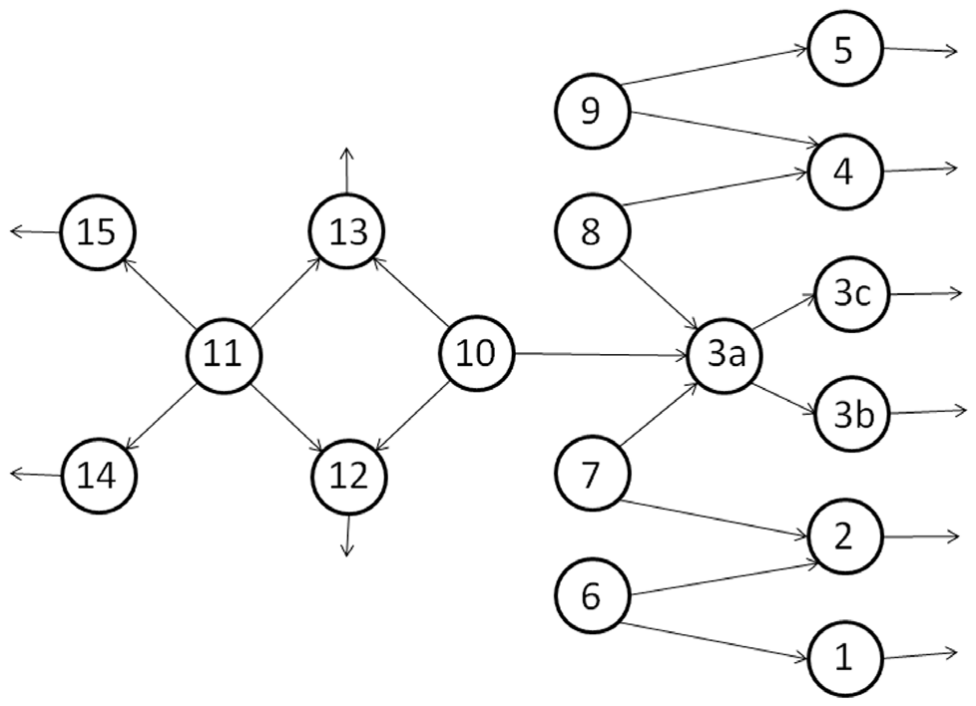
DTSP Network.

**Figure 4:**
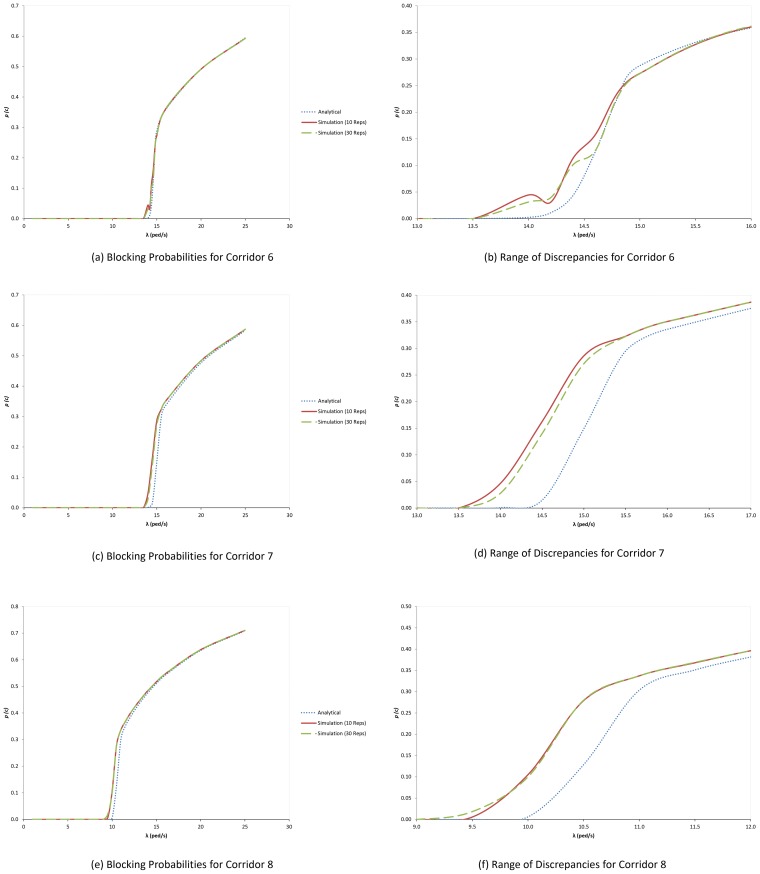
Graph of blocking probabilities measures against variable rates for source corridors. (a) Blocking Probabilities for Corridor 6. (b) Range of Discrepancies for Corridor 6. (c) Blocking Probabilities for Corridor 7. (d) Range of Discrepancies for Corridor 7. (e) Blocking Probabilities for Corridor 8. (f) Range of Discrepancies for Corridor 6.

In the networks under study [4] as shown in Figure 3, there are six source corridors that is Corridors 6 to 11. Figure 4 shows the graph of blocking probabilities measures against variable rates for three of these corridors. There are no blockings until certain points where the blocking probabilities start to increase. For example, for Corridor 7 (Fig. 4(c)), the blocking probability remains zero until blocking starts to appear at about λ≈14 ped/s. However, there are some discrepancies between analytical and simulation results. Figure 4(d) zooms the Fig. 4(c) chart to show that at around the arrival rates 13.5 and 15.5 ped/s, there are significant differences between analytical and simulation (both 10 and 30 replications) results.

For each corridor, there is a range of arrival rates where simulation and animation results exhibit discrepancies. The range could be made smaller if we reduce its blocking probability half width since the blocking probability determine the corridor's throughput, expected service time and expected number of entities. In order to reduce the half width (and thus get a better range of the 95% confidence interval), we have to increase its simulation replication number, *n*. Kelton [15] approximated the minimum number of replications to achieve a relevant expected half width; i.e.:
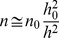



where 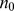 is the number of the initial replication, 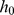 is its half width and 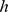 is our expected half width.

We used the formula to find the minimum number of replications that will reduce the half widths of blocking probabilities for all source corridors to less than 5% of their averages. Table 4 shows the range of arrival rates (that simulation and animation results show discrepancies) and their minimum number of replications to achieve the expected half widths for all source corridors.

**Table 4:** Arrival Rates and Their Minimum Number of Replications.

Corridor	Rate	Analytic	Simulation						
			Min	Max	Average	h_0_	Diff (%)	h	n
	14.00	0.0025	0.0000	0.2596	0.0313	0.0304	97.12	0.0016	11319.83
6	14.20	0.0111	0.0000	0.2357	0.0396	0.0278	70.18	0.0020	5909.73
	14.40	0.0437	0.0000	0.2800	0.1013	0.0408	40.31	0.0051	1949.49
	14.60	0.1259	0.0000	0.3019	0.1279	0.0457	35.74	0.0064	1532.72
	14.00	0.0007	0.0000	0.1777	0.0278	0.0208	74.89	0.0014	6730.59
7	14.50	0.0148	0.0000	0.2878	0.1402	0.0443	31.61	0.0070	1199.18
	15.00	0.1494	0.0937	0.3123	0.2714	0.0166	6.12	0.0136	45.00
	15.50	0.2953	0.2979	0.3343	0.3226	0.0035	1.08	0.0161	1.40
	9.50	0.0001	0.0000	0.2004	0.0183	0.0180	98.20	0.0009	11571.12
8	10.00	0.0060	0.0000	0.2671	0.0998	0.0341	34.15	0.0050	1399.33
	10.50	0.1286	0.2123	0.3067	0.2800	0.0093	3.33	0.0140	13.32
	11.00	0.3036	0.3298	0.3430	0.3374	0.0014	0.43	0.0169	0.22
	9.50	0.0001	0.0000	0.2031	0.0309	0.0200	64.82	0.0015	5042.27
	10.00	0.0028	0.0000	0.2544	0.1589	0.0317	19.92	0.0079	476.08
	10.10	0.0052	0.0000	0.2709	0.1963	0.0261	13.32	0.0098	212.79
	10.20	0.0096	0.0184	0.2757	0.2045	0.0216	10.56	0.0102	133.88
	10.30	0.0174	0.1518	0.2833	0.2377	0.0142	5.99	0.0119	43.01
	10.40	0.0303	0.2115	0.2894	0.2675	0.0068	2.55	0.0134	7.80
9	10.50	0.0504	0.2205	0.2962	0.2789	0.0070	2.52	0.0139	7.63
	10.60	0.0791	0.2415	0.3025	0.2947	0.0050	1.71	0.0147	3.49
	10.70	0.1156	0.2703	0.3101	0.3011	0.0040	1.32	0.0151	2.08
	10.80	0.1563	0.2915	0.3156	0.3084	0.0025	0.79	0.0154	0.76
	10.90	0.1963	0.2924	0.3225	0.3191	0.0024	0.74	0.0160	0.65
	11.00	0.2313	0.3162	0.3302	0.3223	0.0016	0.49	0.0161	0.28
	6.40	0.0005	0.0000	0.1439	0.0137	0.0141	102.77	0.0007	12674.93
	6.50	0.0014	0.0000	0.2472	0.0939	0.0372	39.58	0.0047	1880.33
	6.60	0.0039	0.0000	0.2592	0.1222	0.0379	31.03	0.0061	1155.51
10	6.70	0.0101	0.0000	0.2725	0.1989	0.0263	13.23	0.0099	210.13
	6.80	0.0248	0.1266	0.2792	0.2370	0.0162	6.82	0.0119	55.79
	6.90	0.0554	0.1523	0.2924	0.2565	0.0143	5.56	0.0128	37.14
	7.00	0.1068	0.2509	0.3047	0.2891	0.0052	1.80	0.0145	3.89
	6.00	0.0039	0.0000	0.2474	0.1777	0.0191	10.73	0.0089	138.20
	6.10	0.0089	0.0351	0.2596	0.2077	0.0178	8.58	0.0104	88.33
	6.20	0.0193	0.1290	0.2729	0.2291	0.0123	5.37	0.0115	34.59
	6.30	0.0394	0.2190	0.2835	0.2647	0.0059	2.21	0.0132	5.87
	6.40	0.0730	0.2578	0.2945	0.2813	0.0036	1.27	0.0141	1.93
11	6.50	0.1203	0.2679	0.3052	0.2969	0.0029	0.98	0.0148	1.15
	6.60	0.1742	0.2902	0.3190	0.3121	0.0019	0.61	0.0156	0.44
	6.70	0.2246	0.3132	0.3256	0.3212	0.0012	0.38	0.0161	0.17
	6.80	0.2654	0.3238	0.3379	0.3331	0.0012	0.36	0.0167	0.15
	6.90	0.2962	0.3298	0.3464	0.3425	0.0012	0.36	0.0171	0.16
	7.00	0.3193	0.3465	0.3562	0.3524	0.0009	0.27	0.0176	0.09

We can see that for Corridor 6, the blocking probability half widths for 14.00≤λ≤14.60 are so big compared to their averages, since the blocking probabilities fluctuate across replications. For λ = 14.40 pedestrians/second as an example, its minimum blocking probability is 0.0000 while its maximum is 0.2800 with the half width of 0.0408. It is clear that the analytical blocking probability value is located within the minimum and maximum range. If we run our simulation model for 1950 replications, we could decrease the half width to 0.0051 and thus decrease the average of the blocking probability and the simulation results could consistent with the analytical results. However, to run such a big replication number is unpractical and consumes time. Other ranges that the average blocking probabilities could be decreased through the decrease of their half widths are 14.00≤λ≤14.50 (Corridor 7), 9.50≤λ≤10.00 (Corridor 8), 9.50≤λ≤10.30 (Corridor 9), 6.40≤λ≤ 6.80 (Corridor 10) and 6.00≤λ≤6.10 (Corridor 11).

There are ranges where simulation results and analytical results will not ever consistent since their analytical blocking probabilities are not located within the minimum and maximum blocking probabilities. The range are 15.00≤λ≤15.50 (Corridor 7), 10.50≤λ≤11.00 (Corridor 8), 10.40≤λ≤11.00 (Corridor 9), 6.90≤λ≤7.00 (Corridor 10) and 6.30≤λ≤7.00 (Corridor 11). No matter how many replications we run our simulation model, their blocking probability half widths for the ranges will not be significantly reduced.

We can observe that the half widths of the blocking probabilities for Corridor 10 could be decreased if we run our model for 150 replications. For example, λ = 6.80 needs 56 replications to decrease its current half width (that is 0.0162) to its target half width (that is 0.0119). This 150 replication number will also improve other arrival rates. Unfortunately, it will not improve the blocking probability half widths of λ located between 6.90 and 7.00. As a proof of our premises, we ran our simulation model for 150 replications and observed their results. The results of the range of arrival rates and its half widths for Corridor 10 are shown in Table 5.

**Table 5:** Arrival Rates and Half Widths for Corridor 10.

λ	30 replications			150 replication		
	Average	h	Diff (%)	Average	h	Diff (%)
6.4000	0.0137	0.0141	102.9197	0.0339	0.0107	31.5634
6.5000	0.0939	0.0372	39.6166	0.0672	0.0143	21.2798
6.6000	0.1222	0.0379	31.0147	0.1091	0.0147	13.4739
6.7000	0.1989	0.0263	13.2227	0.1738	0.0134	7.7100
6.8000	0.2370	0.0162	6.8354	0.2212	0.0101	4.5660
6.9000	0.2565	0.0143	5.5750	0.2574	0.0063	2.4476
7.0000	0.2891	0.0052	1.7987	0.2815	0.0033	1.1723

We can see that the maximum throughput will happen if arrival rates are 14.00≤λ≤14.42 (Corridor 6), 13.50≤λ≤14.00 (Corridor 7), 9.5≤λ≤ 10.00 (Corridor 8), 9.00≤λ≤9.50 (Corridor 9), 6.20≤λ≤6.40 (Corridor 10) and 5.00≤λ≤ 6.00 (Corridor 11). On the hand, analytical results reported that the maximum throughput will happen when the arrival rates are 14.18 (Corridor 6), 14.46 (Corridor 7), 10.11 (Corridor 8), 10.29 (Corridor 9), 6.75 (Corridor 10) and 6.21 (Corridor 11). Detailed comparisons between analytic and simulation results for all corridors are tabled in Appendices S1, S2, S3, S4, S5, S6.

## Conclusions

We have validated the analytical results of a selected *M/G/C/C* network. From outputs of both models, we observed that the optimal throughput of any corridors happens right before its blocking starts, and the value can be achieved by controlling the arrival rates to the corridor. Smaller arrival rates move pedestrians smoother but cause less throughput at the end. Higher arrival rates meanwhile cause congestion and eventually decrease its final throughput.

Our analysis showed that there is discrepancy between analytical and simulation results on the value of an arrival rate that will cause congestion. However, both models reported almost the same performance measures for arrival rates that are smaller or significantly higher than the value. The results can give ideas on the range of arrival rates that will maximize the throughputs of the source corridors.

As in the analytical model, our simulation model only considers the average travelling distances that pedestrians need to travel from various input sources to exit corridors. However, the exact distance from each source input to the end of corridors needs to be modeled in order to evaluate the real performances of the network so that its results can be used as guidance in an emergency case, e.g. to find the average time to clear the hall. Besides this, the model could be an extremely valuable tool when planning an emergency plan for the network, .e.g. by changing arrival rates to any other distribution types, channeling the flow of pedestrians in the network, etc. Our future researches include embedding animations to our simulation model where decision makers can directly change arrival rates to each of its input sources and see their impacts to pedestrians' behavior and the model's performances. Various performances through graphs, histograms, and tables will help them to get insight into the inner working of the network.

## Supporting Information

Appendix S1 Comparison between Analytic and Simulation for Corridor 6.
(DOCX)

Appendix S2 Comparison between Analytic and Simulation for Corridor 7.
(DOCX)

Appendix S3 Comparison between Analytic and Simulation for Corridor 8.
(DOCX)

Appendix S4 Comparison between Analytic and Simulation for Corridor 9.
(DOCX)

Appendix S5 Comparison between Analytic and Simulation for Corridor 10.
(DOCX)

Appendix S6 Comparison between Analytic and Simulation for Corridor 11.
(DOCX)
