# Supplementary material for: A Discrete Event Simulation Model for Evaluating the Performances of an M/G/C/C State Dependent Queuing System
Source: PLoS One. 2013 Apr 1;8(4):e58402. doi: 10.1371/journal.pone.0058402 (PMC3613361; doi:10.1371/journal.pone.0058402)
Supplement: Appendix S1 — Comparison between Analytic and Simulation for Corridor 6. (DOCX) [file pone.0058402.s001.docx]

**Appendix S1** Comparison between Analytic and Simulation for Corridor 6

| λ | Ө | | p(c) | | L | | W | |
| --- | --- | --- | --- | --- | --- | --- | --- | --- |
|  | Analytic | Simulation | Analytic | Simulation | Analytic | Simulation | Analytic | Simulation |
| 1.00 | 1.0000 | 0.9989  [0.9961, 1.0020] | 0.0000 | 0.0000  [0.0000, 0.0000] | 1.4644 | 1.4628  [1.4590, 1.4670] | 1.4644 | 1.4644  [1.4640, 1.4650] |
| 1.50 | 1.5000 | 1.4988  [1.4960, 1.5020] | 0.0000 | 0.0000  [0.0000, 0.0000] | 2.2189 | 2.2172  [2.2130, 2.2220] | 1.4793 | 1.4793  [1.4790, 1.4790] |
| 2.00 | 2.0000 | 1.9992  [1.9950, 2.0030] | 0.0000 | 0.0000  [0.0000, 0.0000] | 2.9899 | 2.9887  [2.9830, 2.9950] | 1.4949 | 1.4949  [1.4950, 1.4950] |
| 2.50 | 2.5000 | 2.5005  [2.4960, 2.5050] | 0.0000 | 0.0000  [0.0000, 0.0000] | 3.7785 | 3.7793  [3.7720, 3.7870] | 1.5114 | 1.5114  [1.5110, 1.5120] |
| 3.00 | 3.0000 | 3.0056  [3.0010, 3.0100] | 0.0000 | 0.0000  [0.0000, 0.0000] | 4.5860 | 4.5950  [4.5880, 4.6020] | 1.5287 | 1.5288  [1.5290, 1.5290] |
| 3.50 | 3.5000 | 3.5005  [3.4960, 3.5050] | 0.0000 | 0.0000  [0.0000, 0.0000] | 5.4136 | 5.4147  [5.4070, 5.4220] | 1.5468 | 1.5468  [1.5470, 1.5470] |
| 4.00 | 4.0000 | 4.0024  [3.9960, 4.0090] | 0.0000 | 0.0000  [0.0000, 0.0000] | 6.2629 | 6.2668  [6.2560, 6.2780] | 1.5657 | 1.5658  [1.5660, 1.5660] |
| 4.50 | 4.5000 | 4.4985  [4.4930, 4.5040] | 0.0000 | 0.0000  [0.0000, 0.0000] | 7.1353 | 7.1324  [7.1230, 7.1420] | 1.5856 | 1.5855  [1.5850, 1.5860] |
| 5.00 | 5.0000 | 5.0038  [4.9960, 5.0120] | 0.0000 | 0.0000  [0.0000, 0.0000] | 8.0326 | 8.0394  [8.0250, 8.0540] | 1.6065 | 1.6067  [1.6060, 1.6070] |
| 5.50 | 5.5000 | 5.4987  [5.4920, 5.5050] | 0.0000 | 0.0000  [0.0000, 0.0000] | 8.9567 | 8.9543  [8.9420, 8.9660] | 1.6285 | 1.6284  [1.6280, 1.6290] |
| 6.00 | 6.0000 | 6.0011  [5.9950, 6.0070] | 0.0000 | 0.0000  [0.0000, 0.0000] | 9.9096 | 9.9121  [9.9010, 9.9240] | 1.6516 | 1.6517  [1.6510, 1.6520] |
| 6.50 | 6.5000 | 6.4991  [6.4930, 6.5050] | 0.0000 | 0.0000  [0.0000, 0.0000] | 10.8939 | 10.8918  [10.8800, 10.9000] | 1.6760 | 1.6759  [1.6760, 1.6760] |
| 7.00 | 7.0000 | 6.9999  [6.9930, 7.0070] | 0.0000 | 0.0000  [0.0000, 0.0000] | 11.9122 | 11.9122  [11.9000, 11.9300] | 1.7017 | 1.7018  [1.7010, 1.7020] |
| 7.50 | 7.5000 | 7.4908  [7.4820, 7.4990] | 0.0000 | 0.0000  [0.0000, 0.0000] | 12.9675 | 12.9483  [12.9300, 12.9700] | 1.7290 | 1.7286  [1.7280, 1.7290] |
| 8.00 | 8.0000 | 8.0024  [7.9950, 8.0090] | 0.0000 | 0.0000  [0.0000, 0.0000] | 14.0636 | 14.0694  [14.0500, 14.0800] | 1.7579 | 1.7582  [1.7580, 1.7590] |
| 8.50 | 8.5000 | 8.5005  [8.4920, 8.5090] | 0.0000 | 0.0000  [0.0000, 0.0000] | 15.2044 | 15.2060  [15.1900, 15.2300] | 1.7888 | 1.7888  [1.7880, 1.7890] |
| 9.00 | 9.0000 | 8.9975  [8.9890, 9.0060] | 0.0000 | 0.0000  [0.0000, 0.0000] | 16.3949 | 16.3908  [16.3700, 16.4100] | 1.8217 | 1.8217  [1.8210, 1.8220] |
| 9.50 | 9.5000 | 9.4918  [9.4820, 9.5010] | 0.0000 | 0.0000  [0.0000, 0.0000] | 17.6408 | 17.6217  [17.6000, 17.6500] | 1.8569 | 1.8565  [1.8560, 1.8570] |
| 10.00 | 10.0000 | 9.9917  [9.9810, 10.0000] | 0.0000 | 0.0000  [0.0000, 0.0000] | 18.9490 | 18.9287  [18.9000, 18.9600] | 1.8949 | 1.8944  [1.8940, 1.8950] |
| 10.50 | 10.5000 | 10.5017  [10.5000, 10.5100] | 0.0000 | 0.0000  [0.0000, 0.0000] | 20.3279 | 20.3317  [20.3100, 20.3500] | 1.9360 | 1.9360  [1.9350, 1.9370] |
| 11.00 | 11.0000 | 11.0034  [10.9900, 11.0100] | 0.0000 | 0.0000  [0.0000, 0.0000] | 21.7881 | 21.8005  [21.7700, 21.8300] | 1.9807 | 1.9813  [1.9800, 1.9820] |
| 11.50 | 11.5000 | 11.5037  [11.5000, 11.5100] | 0.0000 | 0.0000  [0.0000, 0.0000] | 23.3427 | 23.3564  [23.3400, 23.3800] | 2.0298 | 2.0303  [2.0300, 2.0310] |
| 12.00 | 12.0000 | 11.9935  [11.9800, 12.0000] | 0.0000 | 0.0000  [0.0000, 0.0000] | 25.0090 | 24.9822  [24.9500, 25.0200] | 2.0841 | 2.0830  [2.0820, 2.0840] |
| 12.50 | 12.5000 | 12.4982  [12.4900, 12.5100] | 0.0000 | 0.0000  [0.0000, 0.0000] | 26.8105 | 26.8069  [26.7700, 26.8400] | 2.1448 | 2.1448  [2.1440, 2.1460] |
| 13.00 | 13.0000 | 12.9973  [12.9900, 13.0100] | 0.0000 | 0.0000  [0.0000, 0.0000] | 28.7807 | 28.7705  [28.7400, 28.8000] | 2.2139 | 2.2136  [2.2120, 2.2150] |
| 13.50 | 13.4994 | 13.4960  [13.4900, 13.5100] | 0.0000 | 0.0000  [0.0000, 0.0000] | 30.9914 | 30.9551  [30.9100, 31.0000] | 2.2958 | 2.2936  [2.2920, 2.2960] |
| 14.00 | 13.9652 | 13.5614  [13.1400, 13.9900] | 0.0025 | 0.0313  [0.0009, 0.0617] | 34.4873 | 45.7274  [33.7800, 57.6700] | 2.4695 | 3.6370  [2.4200, 4.8540] |
| 14.20 | 14.0419 | 13.6415  [13.2500, 14.0400] | 0.0111 | 0.0396  [0.0118, 0.0674] | 38.9129 | 49.4758  [39.0400, 59.9100] | 2.7712 | 3.8280  [2.8270, 4.8290] |
| 14.40 | 13.7712 | 12.9373  [12.3500, 13.5200] | 0.0437 | 0.1013  [0.0604, 0.1421] | 51.9059 | 71.9218  [57.3400, 86.5100] | 3.7692 | 6.0293  [4.5300, 7.5280] |
| 14.60 | 12.7616 | 12.7293  [12.0600, 13.4000] | 0.1259 | 0.1279  [0.0822, 0.1736] | 81.2885 | 80.8386  [65.2000, 96.4700] | 6.3698 | 6.9468  [5.3020, 8.5920] |
| 14.80 | 11.4399 | 11.4042  [10.9500,11.8600] | 0.2270 | 0.2288  [0.1976, 0.2601] | 114.4024 | 113.3669  [103.1000, 123.6000] | 10.0003 | 10.2617  [9.1050, 11.4200] |
| 15.00 | 10.6869 | 10.9071  [10.6700,11.1400] | 0.2875 | 0.2726  [0.2570, 0.2882] | 131.6891 | 125.3734  [120.4000, 130.3000] | 12.3225 | 11.5954  [10.9200, 12.2700] |
| 16.00 | 10.2612 | 10.2095  [10.1900,10.2300] | 0.3587 | 0.3615  [0.3603, 0.3627] | 140.0790 | 140.1713  [139.9000, 140.5000] | 13.6514 | 13.7301  [13.6800, 13.7800] |
| 20.00 | 10.1727 | 10.1657  [10.1700,10.1700] | 0.4914 | 0.4917  [0.4914, 0.4914] | 140.9409 | 140.9074  [140.9000, 140.9000] | 13.8547 | 13.8611  [13.8600, 13.8600] |
| 25.00 | 10.1348 | 10.1639  [10.1600,10.1600] | 0.5946 | 0.5931  [0.5929, 0.5933] | 141.3100 | 140.9232  [140.9219, 140.9245] | 13.9430 | 13.8651  [13.8647, 13.8655] |
