# Supplementary material for: A Discrete Event Simulation Model for Evaluating the Performances of an M/G/C/C State Dependent Queuing System
Source: PLoS One. 2013 Apr 1;8(4):e58402. doi: 10.1371/journal.pone.0058402 (PMC3613361; doi:10.1371/journal.pone.0058402)
Supplement: Appendix S2 — Comparison between Analytic and Simulation for Corridor 7. (DOCX) [file pone.0058402.s002.docx]

**Appendix S2** Comparison between Analytic and Simulation for Corridor 7

| λ | Ө | | p(c) | | L | | W | |
| --- | --- | --- | --- | --- | --- | --- | --- | --- |
|  | Analytic | Simulation | Analytic | Simulation | Analytic | Simulation | Analytic | Simulation |
| 1.50 | 1.5000 | 1.5023  [1.4990, 1.5050] | 0.0000 | 0.0000  [0.0000, 0.0000] | 1.8317 | 1.8346  [1.8310, 1.8390] | 1.2212 | 1.2212  [1.2210, 1.2210] |
| 2.00 | 2.0000 | 1.9985  [1.9950, 2.0020] | 0.0000 | 0.0000  [0.0000, 0.0000] | 2.4679 | 2.4659  [2.4620, 2.4700] | 1.2339 | 1.2339  [1.2340, 1.2340] |
| 2.50 | 2.5000 | 2.5006  [2.4970, 2.5050] | 0.0000 | 0.0000  [0.0000, 0.0000] | 3.1184 | 3.1192  [3.1140, 3.1240] | 1.2474 | 1.2474  [1.2470, 1.2480] |
| 3.00 | 3.0000 | 3.0005  [2.9950, 3.0060] | 0.0000 | 0.0000  [0.0000, 0.0000] | 3.7844 | 3.7852  [3.7780, 3.7920] | 1.2615 | 1.2615  [1.2610, 1.2620] |
| 3.50 | 3.5000 | 3.4988  [3.4940, 3.5040] | 0.0000 | 0.0000  [0.0000, 0.0000] | 4.4667 | 4.4650  [4.4580, 4.4720] | 1.2762 | 1.2761  [1.2760, 1.2760] |
| 4.00 | 4.0000 | 4.0020  [3.9980, 4.0060] | 0.0000 | 0.0000  [0.0000, 0.0000] | 5.1665 | 5.1691  [5.1630, 5.1750] | 1.2916 | 1.2916  [1.2910, 1.2920] |
| 4.50 | 4.5000 | 4.5008  [4.4950, 4.5070] | 0.0000 | 0.0000  [0.0000, 0.0000] | 5.8852 | 5.8868  [5.8780, 5.8960] | 1.3078 | 1.3079  [1.3080, 1.3080] |
| 5.00 | 5.0000 | 5.0000  [4.9950, 5.0060] | 0.0000 | 0.0000  [0.0000, 0.0000] | 6.6240 | 6.6243  [6.6160, 6.6320] | 1.3248 | 1.3249  [1.3250, 1.3250] |
| 5.50 | 5.5000 | 5.5034  [5.4980, 5.5090] | 0.0000 | 0.0000  [0.0000, 0.0000] | 7.3845 | 7.3897  [7.3810, 7.3980] | 1.3426 | 1.3428  [1.3430, 1.3430] |
| 6.00 | 6.0000 | 5.9988  [5.9910, 6.0060] | 0.0000 | 0.0000  [0.0000, 0.0000] | 8.1683 | 8.1663  [8.1540, 8.1780] | 1.3614 | 1.3613  [1.3610, 1.3620] |
| 6.50 | 6.5000 | 6.5038  [6.4970, 6.5110] | 0.0000 | 0.0000  [0.0000, 0.0000] | 8.9774 | 8.9831  [8.9720, 8.9950] | 1.3811 | 1.3812  [1.3810, 1.3820] |
| 7.00 | 7.0000 | 7.0015  [6.9950, 7.0080] | 0.0000 | 0.0000  [0.0000, 0.0000] | 9.8139 | 9.8159  [9.8050, 9.8260] | 1.4020 | 1.4020  [1.4020, 1.4020] |
| 7.50 | 7.5000 | 7.5005  [7.4930, 7.5080] | 0.0000 | 0.0000  [0.0000, 0.0000] | 10.6802 | 10.6806  [10.6700, 10.6900] | 1.4240 | 1.4240  [1.4240, 1.4240] |
| 8.00 | 8.0000 | 7.9961  [7.9900, 8.0020] | 0.0000 | 0.0000  [0.0000, 0.0000] | 11.5791 | 11.5713  [11.5600, 11.5800] | 1.4474 | 1.4471  [1.4470, 1.4470] |
| 8.50 | 8.5000 | 8.5011  [8.4930, 8.5090] | 0.0000 | 0.0000  [0.0000, 0.0000] | 12.5139 | 12.5166  [12.5000, 12.5300] | 1.4722 | 1.4723  [1.4720, 1.4730] |
| 9.00 | 9.0000 | 8.9969  [8.9880, 9.0060] | 0.0000 | 0.0000  [0.0000, 0.0000] | 13.4885 | 13.4823  [13.4600, 13.5000] | 1.4987 | 1.4985  [1.4980, 1.4990] |
| 9.50 | 9.5000 | 9.4977  [9.4900, 9.5060] | 0.0000 | 0.0000  [0.0000, 0.0000] | 14.5071 | 14.5019  [14.4800, 14.5200] | 1.5271 | 1.5269  [1.5260, 1.5270] |
| 10.00 | 10.0000 | 9.9994  [9.9910, 10.0100] | 0.0000 | 0.0000  [0.0000, 0.0000] | 15.5753 | 15.5749  [15.5600, 15.5900] | 1.5575 | 1.5576  [1.5570, 1.5580] |
| 10.50 | 10.5000 | 10.4951  [10.4900, 10.5000] | 0.0000 | 0.0000  [0.0000, 0.0000] | 16.6995 | 16.6868  [16.6700, 16.7100] | 1.5904 | 1.5900  [1.5890, 1.5910] |
| 11.00 | 11.0000 | 11.0011  [10.9900, 11.0100] | 0.0000 | 0.0000  [0.0000, 0.0000] | 17.8877 | 17.8907  [17.8700, 17.9100] | 1.6262 | 1.6263  [1.6260, 1.6270] |
| 11.50 | 11.5000 | 11.4987  [11.4900, 11.5100] | 0.0000 | 0.0000  [0.0000, 0.0000] | 19.1501 | 19.1475  [19.1300, 19.1700] | 1.6652 | 1.6652  [1.6640,1.6660] |
| 12.00 | 12.0000 | 11.9937  [11.9800, 12.0000] | 0.0000 | 0.0000  [0.0000, 0.0000] | 20.4999 | 20.4837  [20.4600, 20.5100] | 1.7083 | 1.7079  [1.7070,1.7090] |
| 12.50 | 12.5000 | 12.4936  [12.4900, 12.5000] | 0.0000 | 0.0000  [0.0000, 0.0000] | 21.9545 | 21.9379  [21.9100, 21.9600] | 1.7564 | 1.7559  [1.7550,1.7570] |
| 13.00 | 13.0000 | 12.9996  [12.9900, 13.0100] | 0.0000 | 0.0000  [0.0000, 0.0000] | 23.5392 | 23.5419  [23.5100, 23.5700] | 1.8107 | 1.8110  [1.8100,1.8120] |
| 13.50 | 13.4997 | 13.5056  [13.4900, 13.5200] | 0.0000 | 0.0000  [0.0000, 0.0000] | 25.3009 | 25.3085  [25.2700, 25.3500] | 1.8742 | 1.8739  [1.8720,1.8750] |
| 14.00 | 13.9909 | 13.6133  [13.3200, 13.9000] | 0.0007 | 0.0278  [0.0070, 0.0487] | 27.5260 | 37.0234  [29.7300, 44.3100] | 1.9674 | 2.8174  [2.1570,3.4780] |
| 14.50 | 14.2849 | 12.4592  [11.8200, 13.1000] | 0.0148 | 0.1402  [0.0959, 0.1845] | 34.5432 | 73.1561  [59.3800, 86.9300] | 2.4182 | 6.3846  [4.9430,7.8260] |
| 15.00 | 12.7589 | 10.9265  [10.6800, 11.1800] | 0.1494 | 0.2714  [0.2548, 0.2880] | 75.9530 | 107.7527  [103.1000, 112.4000] | 5.9529 | 9.9536  [9.3720,10.5400] |
| 15.50 | 10.9228 | 10.4943  [10.4400, 10.5500] | 0.2953 | 0.3226  [0.3191, 0.3261] | 111.7883 | 116.2016  [115.3000, 117.1000] | 10.2344 | 11.0777  [10.9400,11.2100] |
| 16.00 | 10.6248 | 10.3778  [10.3600, 10.3900] | 0.3359 | 0.3508  [0.3497, 0.3519] | 116.4431 | 118.1701  [117.9000, 118.4000] | 10.9596 | 11.3873  [11.3500,11.4300] |
| 20.00 | 10.4674 | 10.3266  [10.3300, 10.3300] | 0.4766 | 0.4835  [0.4832, 0.4838] | 117.8681 | 118.9262  [118.9000, 118.9000] | 11.2605 | 11.5165  [11.5200,11.5200] |
| 25.00 | 10.4166 | 10.3252  [10.3300, 10.3300] | 0.5833 | 0.5867  [0.5865, 0.5869] | 118.27468 | 118.9515  [118.9000, 119.0000] | 11.3543 | 11.5205  [11.5200,11.5200] |
