# Supplementary material for: A Discrete Event Simulation Model for Evaluating the Performances of an M/G/C/C State Dependent Queuing System
Source: PLoS One. 2013 Apr 1;8(4):e58402. doi: 10.1371/journal.pone.0058402 (PMC3613361; doi:10.1371/journal.pone.0058402)
Supplement: Appendix S3 — Comparison between Analytic and Simulation for Corridor 8. (DOCX) [file pone.0058402.s003.docx]

**Appendix S3** Comparison between Analytic and Simulation for Corridor 8

| λ | Ө | | p(c) | | L | | W | |
| --- | --- | --- | --- | --- | --- | --- | --- | --- |
|  | Analytic | Simulation | Analytic | Simulation | Analytic | Simulation | Analytic | Simulation |
| 1.00 | 1.0000 | 0.9986  [0.9958, 1.0010] | 0.0000 | 0.0000  [0.0000, 0.0000] | 1.4776 | 1.4755  [1.4710, 1.4800] | 1.4776 | 1.4776  [1.4770, 1.4780] |
| 1.50 | 1.5000 | 1.4994  [1.4960, 1.5030] | 0.0000 | 0.0000  [0.0000, 0.0000] | 2.2502 | 2.2491  [2.2440, 2.2540] | 1.5001 | 1.5  [1.5000, 1.5000] |
| 2.00 | 2.0000 | 2.0007  [1.9970, 2.0040] | 0.0000 | 0.0000  [0.0000, 0.0000] | 3.0484 | 3.0498  [3.0440, 3.0560] | 1.5242 | 1.5243  [1.5240, 1.5250] |
| 2.50 | 2.5000 | 2.5010  [2.4970, 2.5050] | 0.0000 | 0.0000  [0.0000, 0.0000] | 3.8749 | 3.8767  [3.8700, 3.8830] | 1.5500 | 1.5501  [1.5500, 1.5500] |
| 3.00 | 3.0000 | 3.0016  [2.9980, 3.0060] | 0.0000 | 0.0000  [0.0000, 0.0000] | 4.7324 | 4.7348  [4.7280, 4.7420] | 1.5775 | 1.5774  [1.5770, 1.5780] |
| 3.50 | 3.5000 | 3.4982  [3.4940, 3.5030] | 0.0000 | 0.0000  [0.0000, 0.0000] | 5.6241 | 5.6213  [5.6130, 5.6290] | 1.6069 | 1.6069  [1.6070, 1.6070] |
| 4.00 | 4.0000 | 3.9998  [3.9950, 4.0050] | 0.0000 | 0.0000  [0.0000, 0.0000] | 6.5539 | 6.5537  [6.5440, 6.5640] | 1.6385 | 1.6385  [1.6380, 1.6390] |
| 4.50 | 4.5000 | 4.4998  [4.4940, 4.5060] | 0.0000 | 0.0000  [0.0000, 0.0000] | 7.5261 | 7.5263  [7.5150, 7.5380] | 1.6725 | 1.6726  [1.6720, 1.6730] |
| 5.00 | 5.0000 | 4.9982  [4.9930, 5.0040] | 0.0000 | 0.0000  [0.0000, 0.0000] | 8.5459 | 8.5430  [8.5310, 8.5550] | 1.7092 | 1.7092  [1.7090, 1.7100] |
| 5.50 | 5.5000 | 5.5063  [5.4990, 5.5140] | 0.0000 | 0.0000  [0.0000, 0.0000] | 9.6196 | 9.6335  [9.6180, 9.6490] | 1.7490 | 1.7495  [1.7490, 1.7500] |
| 6.00 | 6.0000 | 6.0016  [5.9940, 6.0090] | 0.0000 | 0.0000  [0.0000, 0.0000] | 10.7548 | 10.7591  [10.7400, 10.7800] | 1.7925 | 1.7927  [1.7920, 1.7930] |
| 6.50 | 6.5000 | 6.5035  [6.4980, 6.5090] | 0.0000 | 0.0000  [0.0000, 0.0000] | 11.9614 | 11.9697  [11.9600, 11.9800] | 1.8402 | 1.8405  [1.8400, 1.8410] |
| 7.00 | 7.0000 | 6.9983  [6.9910, 7.0060] | 0.0000 | 0.0000  [0.0000, 0.0000] | 13.2515 | 13.251  [13.2300, 13.2700] | 1.8931 | 1.8934  [1.8920, 1.8940] |
| 7.50 | 7.5000 | 7.4980  [7.4910, 7.5050] | 0.0000 | 0.0000  [0.0000, 0.0000] | 14.6415 | 14.6362  [14.6200, 14.6600] | 1.9522 | 1.952  [1.9510, 1.9530] |
| 8.00 | 8.0000 | 7.9999  [7.9920, 8.0080] | 0.0000 | 0.0000  [0.0000, 0.0000] | 16.1536 | 16.1534  [16.1300, 16.1800] | 2.0192 | 2.0192  [2.0180, 2.0200] |
| 8.50 | 8.8000 | 8.4981  [8.4920, 8.5040] | 0.0000 | 0.0000  [0.0000, 0.0000] | 18.9137 | 17.8104  [17.7900, 17.8300] | 2.1493 | 2.0958  [2.0950, 2.0970] |
| 9.00 | 9.0000 | 9.0007  [8.9920, 9.0100] | 0.0000 | 0.0000  [0.0000, 0.0000] | 19.6917 | 19.6917  [19.6500, 19.7300] | 2.1880 | 2.1878  [2.1860, 2.1900] |
| 9.50 | 9.4989 | 9.3204  [9.1500, 9.4910] | 0.0001 | 0.0183  [0.0003, 0.0363] | 21.9006 | 27.9327  [21.9700, 33.9000] | 2.3056 | 3.0991  [2.2920, 3.9060] |
| 10.00 | 9.9399 | 8.9933  [8.6530, 9.3330] | 0.0060 | 0.0998  [0.0657, 0.1339] | 26.3468 | 52.0894  [42.6500, 61.5300] | 2.6506 | 6.141  [4.8040, 7.4780] |
| 10.50 | 9.1496 | 7.5503  [7.4550, 7.6460] | 0.1286 | 0.2800  [0.2707, 0.2893] | 60.0732 | 93.8997  [91.7300, 96.0700] | 6.5657 | 12.4745  [12.0400, 12.9100] |
| 11.00 | 7.6604 | 7.2831  [7.2690, 7.2970] | 0.3036 | 0.3374  [0.3360, 0.3388] | 95.4085 | 100.1245  [99.8500, 100.4000] | 12.4548 | 13.7483  [13.6800, 13.8100] |
| 11.50 | 7.4626 | 7.2560  [7.2510, 7.2610] | 0.3511 | 0.3688  [0.3681, 0.3696] | 98.7775 | 100.6675  [100.6000, 100.7000] | 13.2364 | 13.8737  [13.8500, 13.8900] |
| 12.00 | 7.4218 | 7.2448  [7.2420, 7.2470] | 0.3815 | 0.3961  [0.3957, 0.3965] | 99.2339 | 100.8539  [100.8000, 100.9000] | 13.3706 | 13.9208  [13.9100, 13.9300] |
| 12.50 | 7.3990 | 7.2439  [7.2430, 7.2450] | 0.4081 | 0.4203  [0.4198, 0.4207] | 99.4560 | 100.8687  [100.8000, 100.9000] | 13.4417 | 13.9247  [13.9200, 13.9300] |
| 13.00 | 7.3823 | 7.2421  [7.2410, 7.2430] | 0.4321 | 0.4424  [0.4420, 0.4428] | 99.6176 | 100.8974  [100.9000, 100.9000] | 13.4941 | 13.9321  [13.9300, 13.9400] |
| 13.50 | 7.3691 | 7.2408  [7.2400, 7.2420] | 0.4541 | 0.4637  [0.4633, 0.4641] | 99.7456 | 100.9154  [100.9000, 100.9000] | 13.5357 | 13.937  [13.9300, 13.9400] |
| 14.00 | 7.3582 | 7.2402  [7.2400, 7.2410] | 0.4744 | 0.4825  [0.4822, 0.4828] | 99.8505 | 100.9251  [100.9000, 100.9000] | 13.5699 | 13.9395  [13.9400, 13.9400] |
| 14.50 | 7.3491 | 7.2396  [7.2390, 7.2400] | 0.4932 | 0.5002  [0.4998, 0.5005] | 99.9384 | 100.9323  [100.9000, 100.9000] | 13.5987 | 13.9418  [13.9400, 13.9400] |
| 15.00 | 7.3414 | 7.2394  [7.2390, 7.2400] | 0.5106 | 0.5172  [0.5169, 0.5175] | 100.0133 | 100.9362  [100.9000, 100.9000] | 13.6232 | 13.9427  [13.9400, 13.9400] |
| 16.00 | 7.3289 | 7.2393  [7.2390, 7.2390] | 0.5419 | 0.5471  [0.5468, 0.5474] | 100.1346 | 100.9440  [100.9000, 100.9000] | 13.6630 | 13.9439  [13.9400, 13.9400] |
| 20.00 | 7.2998 | 7.2386  [7.2380, 7.2390] | 0.6350 | 0.6377  [0.6376, 0.6379] | 100.4176 | 100.9526  [101.0000, 101.0000] | 13.7562 | 13.9464  [13.9500, 13.9500] |
| 25.00 | 7.2826 | 7.2380  [7.2380, 7.2380] | 0.7087 | 0.7103  [0.7101, 0.7104] | 100.5855 | 100.9536  [101.0000, 101.0000] | 13.8118 | 13.9477  [13.9500, 13.9500] |
