# Supplementary material for: A Discrete Event Simulation Model for Evaluating the Performances of an M/G/C/C State Dependent Queuing System
Source: PLoS One. 2013 Apr 1;8(4):e58402. doi: 10.1371/journal.pone.0058402 (PMC3613361; doi:10.1371/journal.pone.0058402)
Supplement: Appendix S4 — Comparison between Analytic and Simulation for Corridor 9. (DOCX) [file pone.0058402.s004.docx]

**Appendix S4** Comparison between Analytic and Simulation for Corridor 9

| λ | Ө | | p(c) | | L | | W | |
| --- | --- | --- | --- | --- | --- | --- | --- | --- |
|  | Analytic | Simulation | Analytic | Simulation | Analytic | Simulation | Analytic | Simulation |
| 1.00 | 1.0000 | 1.0021  [0.9993, 1.0050] | 0.0000 | 0.0000  [0.0000, 0.0000] | 1.2200 | 1.2227  [1.2190, 1.2260] | 1.2200 | 1.2201  [1.2200, 1.2200] |
| 1.50 | 1.5000 | 1.4998  [1.4970, 1.5030] | 0.0000 | 0.0000  [0.0000, 0.0000] | 1.8577 | 1.8575  [1.8540, 1.8610] | 1.2385 | 1.2385  [1.2380, 1.2390] |
| 2.00 | 2.0000 | 1.9984  [1.9940, 2.0030] | 0.0000 | 0.0000  [0.0000, 0.0000] | 2.5165 | 2.5144  [2.5090, 2.5200] | 1.2582 | 1.2582  [1.2580, 1.2580] |
| 2.50 | 2.5000 | 2.4997  [2.4940, 2.5050] | 0.0000 | 0.0000  [0.0000, 0.0000] | 3.1982 | 3.1981  [3.1910, 3.2050] | 1.2793 | 1.2794  [1.2790, 1.2800] |
| 3.00 | 3.0000 | 2.9973  [2.9920, 3.0020] | 0.0000 | 0.0000  [0.0000, 0.0000] | 3.9054 | 3.9014  [3.8940, 3.9080] | 1.3018 | 1.3016  [1.3010, 1.3020] |
| 3.50 | 3.5000 | 3.5015  [3.4970, 3.5060] | 0.0000 | 0.0000  [0.0000, 0.0000] | 4.6404 | 4.6427  [4.6360, 4.6490] | 1.3258 | 1.3259  [1.3260, 1.3260] |
| 4.00 | 4.0000 | 4.0026  [3.9970, 4.0080] | 0.0000 | 0.0000  [0.0000, 0.0000] | 5.4064 | 5.4104  [5.4020, 5.4190] | 1.3516 | 1.3517  [1.3510, 1.3520] |
| 4.50 | 4.5000 | 4.5003  [4.4950, 4.5060] | 0.0000 | 0.0000  [0.0000, 0.0000] | 6.2068 | 6.2078  [6.1980, 6.2170] | 1.3793 | 1.3794  [1.3790, 1.3800] |
| 5.00 | 5.0000 | 5.0039  [4.9970, 5.0100] | 0.0000 | 0.0000  [0.0000, 0.0000] | 7.0459 | 7.0524  [7.0410, 7.0630] | 1.4092 | 1.4094  [1.4090, 1.4100] |
| 5.50 | 5.5000 | 5.5013  [5.4950, 5.5070] | 0.0000 | 0.0000  [0.0000, 0.0000] | 7.9285 | 7.9304  [7.9200, 7.9410] | 1.4415 | 1.4416  [1.4410, 1.4420] |
| 6.00 | 6.0000 | 6.0003  [5.9910, 6.0100] | 0.0000 | 0.0000  [0.0000, 0.0000] | 8.8609 | 8.8623  [8.8440, 8.8800] | 1.4768 | 1.477  [1.4760, 1.4780] |
| 6.50 | 6.5000 | 6.4930  [6.4880, 6.4980] | 0.0000 | 0.0000  [0.0000, 0.0000] | 9.8507 | 9.8363  [9.8250, 9.8480] | 1.5155 | 1.5149  [1.5140, 1.5150] |
| 7.00 | 7.0000 | 7.0008  [6.9960, 7.0050] | 0.0000 | 0.0000  [0.0000, 0.0000] | 10.9076 | 10.9079  [10.9000, 10.9200] | 1.5582 | 1.5581  [1.5580, 1.5590] |
| 7.50 | 7.5000 | 7.4953  [7.4900, 7.5010] | 0.0000 | 0.0000  [0.0000, 0.0000] | 12.0445 | 12.0335  [12.0200, 12.0500] | 1.6059 | 1.6055  [1.6050, 1.6060] |
| 8.00 | 8.0000 | 7.9960  [7.9880, 8.0040] | 0.0000 | 0.0000  [0.0000, 0.0000] | 13.2789 | 13.2687  [13.2500, 13.2900] | 1.6599 | 1.6594  [1.6580, 1.6600] |
| 8.50 | 8.5000 | 8.4978  [8.4890, 8.5070] | 0.0000 | 0.0000  [0.0000, 0.0000] | 14.6359 | 14.6330  [14.6100, 14.6600] | 1.7219 | 1.722  [1.7210, 1.7230] |
| 9.00 | 9.0000 | 8.9578  [8.8780, 9.0380] | 0.0000 | 0.0044  [-0.0046, 0.0134] | 16.1561 | 17.8141  [14.4100, 21.2200] | 1.7951 | 2.0166  [1.5630, 2.4700] |
| 9.50 | 9.4991 | 9.2524  [9.0620, 9.4430] | 0.0001 | 0.0261  [0.0061, 0.0462] | 17.9404 | 25.7982  [19.7400, 31.8500] | 1.8886 | 2.903  [2.0970, 3.7090] |
| 10.00 | 9.9723 | 8.5768  [8.2580, 8.8960] | 0.0028 | 0.1425  [0.1109, 0.1742] | 20.8172 | 55.4031  [47.5400, 63.2700] | 2.0875 | 6.7508  [5.6160, 7.8850] |
| 10.10 | 10.0473 | 8.1395  [7.8750, 8.4040] | 0.0052 | 0.1943  [0.1682, 0.2204] | 21.9372 | 67.0588  [60.7800, 73.3400] | 2.1834 | 8.4595  [7.4770, 9.4420] |
| 10.20 | 10.1017 | 7.9238  [7.7030, 8.1440] | 0.0096 | 0.2226  [0.2010, 0.2442] | 23.5420 | 72.6273  [67.5900, 77.6600] | 2.3305 | 9.3199  [8.5170, 10.1200] |
| 10.29 | 10.1213 | 7.7728  [7.6260, 7.9200] | 0.0164 | 0.2450  [0.2307, 0.2592] | 25.6258 | 76.5278  [73.3400, 79.7200] | 2.531863 | 9.9201  [9.3520, 10.4900] |
| 10.30 | 10.1211 | 7.8381  [7.5710, 8.1050] | 0.0174 | 0.2378  [0.2117, 0.2640] | 25.9060 | 74.9283  [69.0200, 80.8300] | 2.5596 | 9.7677  [8.8810, 10.6500] |
| 10.40 | 10.0853 | 7.6011  [7.5300, 7.6720] | 0.0303 | 0.2689  [0.2621, 0.2757] | 29.3805 | 80.4339  [78.9300, 81.9400] | 2.9132 | 10.6005  [10.3100, 10.8900] |
| 10.50 | 9.9710 | 7.5631  [7.4870, 7.6400] | 0.0504 | 0.2794  [0.2724, 0.2865] | 34.3058 | 81.3844  [79.8500, 82.9200] | 3.4406 | 10.7816  [10.4800, 11.0800] |
| 10.60 | 9.7616 | 7.5164  [7.4630, 7.5700] | 0.0791 | 0.2906  [0.2856, 0.2956] | 40.8016 | 82.4112  [81.3600, 83.4700] | 4.1798 | 10.9746  [10.7600, 11.1900] |
| 10.70 | 9.4630 | 7.5036  [7.4620, 7.5450] | 0.1156 | 0.2983  [0.2943, 0.3022] | 48.5116 | 82.7232  [81.9400, 83.5100] | 5.1264 | 11.0306  [10.8700, 11.1900] |
| 10.80 | 9.1114 | 7.4623  [7.4350, 7.4900] | 0.1563 | 0.3083  [0.3059, 0.3108] | 56.5589 | 83.5661  [83.0800, 84.0500] | 6.2075 | 11.2011  [11.1000, 11.3100] |
| 10.90 | 8.7606 | 7.4402  [7.4150, 7.4650] | 0.1963 | 0.3171  [0.3147, 0.3195] | 63.9038 | 84.0201  [83.6000, 84.4400] | 7.2945 | 11.2949  [11.2000, 11.3900] |
| 11.00 | 8.4552 | 7.4405  [7.4250, 7.4560] | 0.2313 | 0.3234  [0.3218, 0.3249] | 69.8442 | 84.0143  [83.7500, 84.2800] | 8.2605 | 11.2923  [11.2300, 11.3500] |
| 12.00 | 7.6419 | 7.3943  [7.3910, 7.3970] | 0.3632 | 0.3830  [0.3824, 0.3835] | 82.9006 | 84.8321  [84.7900, 84.8700] | 10.8481 | 11.4727  [11.4600, 11.4800] |
| 13.00 | 7.5723 | 7.3872  [7.3860, 7.3880] | 0.4175 | 0.4315  [0.4312, 0.4319] | 83.5001 | 84.9284  [84.9200, 84.9400] | 11.0270 | 11.4967  [11.4900, 11.5000] |
| 14.00 | 7.5380 | 7.3863  [7.3860, 7.3870] | 0.4616 | 0.4719  [0.4716, 0.4723] | 83.7743 | 84.9402  [84.9300, 84.9500] | 11.1136 | 11.4997  [11.5000, 11.5000] |
| 15.00 | 7.5151 | 7.3851  [7.3850, 7.3850] | 0.4990 | 0.5072  [0.5069, 0.5075] | 83.9574 | 84.9545  [84.9500, 84.9600] | 11.1719 | 11.5035  [11.5000, 11.5000] |
| 20.00 | 7.4605 | 7.3840  [7.3840, 7.3840] | 0.6270 | 0.6305  [0.6303, 0.6307] | 84.3951 | 84.9653  [84.9600, 84.9700] | 11.3123 | 11.5066  [11.5100, 11.5100] |
| 25.00 | 7.4385 | 7.3837  [7.3840, 7.3840] | 0.7025 | 0.7045  [0.7043, 0.7046] | 84.5720 | 84.9665  [84.9700, 84.9700] | 11.36948 | 11.5073  [11.5100, 11.5100] |
