# Supplementary material for: A Discrete Event Simulation Model for Evaluating the Performances of an M/G/C/C State Dependent Queuing System
Source: PLoS One. 2013 Apr 1;8(4):e58402. doi: 10.1371/journal.pone.0058402 (PMC3613361; doi:10.1371/journal.pone.0058402)
Supplement: Appendix S5 — Comparison between Analytic and Simulation for Corridor 10. (DOCX) [file pone.0058402.s005.docx]

**Appendix S5** Comparison between Analytic and Simulation for Corridor 10

| λ | Ө | | p(c) | | L | | W | |
| --- | --- | --- | --- | --- | --- | --- | --- | --- |
|  | Analytic | Simulation | Analytic | Simulation | Analytic | Simulation | Analytic | Simulation |
| 1.00 | 1.0000 | 1.0003  [0.9972, 1.0030] | 0.0000 | 0.0000  [0.0000, 0.0000] | 1.8795 | 1.8800  [1.8740, 1.8860] | 1.8795 | 1.8795  [1.8790, 1.8800] |
| 1.50 | 1.5000 | 1.5015  [1.4980, 1.5050] | 0.0000 | 0.0000  [0.0000, 0.0000] | 2.8887 | 2.8919  [2.8850, 2.8990] | 1.9258 | 1.9260  [1.9260, 1.9260] |
| 2.00 | 2.0000 | 2.0021  [1.9990, 2.0050] | 0.0000 | 0.0000  [0.0000, 0.0000] | 3.9537 | 3.9584  [3.9510, 3.9660] | 1.9769 | 1.9771  [1.9770, 1.9780] |
| 2.50 | 2.5000 | 2.5000  [2.4960, 2.5040] | 0.0000 | 0.0000  [0.0000, 0.0000] | 5.0835 | 5.0840  [5.0740, 5.0940] | 2.0334 | 2.0336  [2.0330, 2.0340] |
| 3.00 | 3.0000 | 2.9978  [2.9940, 3.0020] | 0.0000 | 0.0000  [0.0000, 0.0000] | 6.2891 | 6.2834  [6.2730, 6.2930] | 2.0964 | 2.0960  [2.0950, 2.0970] |
| 3.50 | 3.5000 | 3.4995  [3.4960, 3.5030] | 0.0000 | 0.0000  [0.0000, 0.0000] | 7.5846 | 7.5836  [7.5730, 7.5940] | 2.1670 | 2.1670  [2.1660, 2.1680] |
| 4.00 | 4.0000 | 4.0047  [3.9990, 4.0110] | 0.0000 | 0.0000  [0.0000, 0.0000] | 8.9891 | 9.0007  [8.9830, 9.0190] | 2.2473 | 2.2475  [2.2460, 2.2490] |
| 4.50 | 4.5000 | 4.4994  [4.4940, 4.5050] | 0.0000 | 0.0000  [0.0000, 0.0000] | 10.5286 | 10.5269  [10.5100, 10.5400] | 2.3397 | 2.3396  [2.3380, 2.3410] |
| 5.00 | 5.0000 | 5.0016  [4.9960, 5.0070] | 0.0000 | 0.0000  [0.0000, 0.0000] | 12.2414 | 12.2470  [12.2300, 12.2700] | 2.4483 | 2.4486  [2.4470, 2.4500] |
| 5.50 | 5.5000 | 5.4989  [5.4930, 5.5040] | 0.0000 | 0.0000  [0.0000, 0.0000] | 14.1882 | 14.1830  [14.1600, 14.2100] | 2.5797 | 2.5792  [2.5770, 2.5810] |
| 6.00 | 6.0000 | 5.9986  [5.9920, 6.0050] | 0.0000 | 0.0000  [0.0000, 0.0000] | 16.4811 | 16.4785  [16.4400, 16.5100] | 2.7469 | 2.7470  [2.7440, 2.7500] |
| 6.10 | 6.0999 | 6.0986  [6.0920, 6.1060] | 0.0000 | 0.0000  [0.0000, 0.0000] | 17.0028 | 16.9848  [16.9500, 17.0200] | 2.7874 | 2.7850  [2.7820, 2.7880] |
| 6.20 | 6.1996 | 6.1806  [6.1480, 6.2130] | 0.0001 | 0.0025  [-0.0027, 0.0077] | 17.5592 | 18.3160  [16.6800, 19.9600] | 2.8323 | 2.9742  [2.6720, 3.2760] |
| 6.30 | 6.2989 | 6.1147  [5.9530, 6.2770] | 0.0002 | 0.0290  [0.0032, 0.0548] | 18.1693 | 26.6664  [19.0300, 34.3000] | 2.8845 | 4.6561  [3.0460, 6.2660] |
| 6.40 | 6.3968 | 6.3091  [6.2190, 6.3990] | 0.0005 | 0.0137  [-0.0004, 0.0278] | 18.8764 | 22.5176  [18.6200, 26.4100] | 2.9509 | 3.6424  [2.8980, 4.3860] |
| 6.50 | 6.4908 | 5.8886  [5.6460, 6.1310] | 0.0014 | 0.0939  [0.0567, 0.1310] | 19.7816 | 44.0060  [34.2400, 53.7700] | 3.0476 | 8.0682  [5.9800, 10.1600] |
| 6.60 | 6.5745 | 5.7897  [5.5420, 6.0380] | 0.0039 | 0.1222  [0.0842, 0.1601] | 21.1183 | 50.3348  [40.9200, 59.7500] | 3.2121 | 9.2968  [7.2620, 11.3300] |
| 6.70 | 6.6324 | 5.3610  [5.1870, 5.5350] | 0.0101 | 0.1989  [0.1726, 0.2252] | 23.3958 | 67.6121  [61.3700, 73.8500] | 3.5275 | 12.9320  [11.5000, 14.3600] |
| 6.80 | 6.6314 | 5.1855  [5.0780, 5.2930] | 0.0248 | 0.2370  [0.2208, 0.2531] | 27.5855 | 74.6467  [71.0700, 78.2300] | 4.1598 | 14.5289  [13.6000, 15.4600] |
| 6.90 | 6.5180 | 5.1261  [5.0290, 5.2230] | 0.0554 | 0.2565  [0.2423, 0.2708] | 35.0239 | 77.0752  [74.0400, 80.1100] | 5.3734 | 15.1452  [14.3100, 15.9800] |
| 7.00 | 6.2524 | 4.9705  [4.9340, 5.0070] | 0.1068 | 0.2891  [0.2839, 0.2944] | 46.2203 | 82.1348  [81.0700, 83.2000] | 7.3924 | 16.5411  [16.2100, 16.8700] |
| 8.00 | 4.9440 | 4.8832  [4.8810, 4.8850] | 0.3820 | 0.3892  [0.3885, 0.3899] | 84.1638 | 84.7699  [84.7300, 84.8100] | 17.0233 | 17.3595  [17.3400, 17.3800] |
| 9.00 | 4.8977 | 4.8762  [4.8760, 4.8770] | 0.4558 | 0.4571  [0.4568, 0.4575] | 84.7419 | 84.9167  [84.9100, 84.9200] | 17.3024 | 17.4146  [17.4100, 17.4200] |
| 10.00 | 4.8753 | 4.8753  [4.8750, 4.8760] | 0.5125 | 0.5121  [0.5116, 0.5125] | 85.0150 | 84.9289  [84.9300, 84.9300] | 17.4380 | 17.4203  [17.4200, 17.4200] |
| 15.00 | 4.8342 | 4.8744  [4.8740, 4.8740] | 0.6777 | 0.6747  [0.6745, 0.6749] | 85.5186 | 84.9521  [84.9500, 84.9500] | 17.6904 | 17.4283  [17.4300, 17.4300] |
| 20.00 | 4.821067 | 4.8749  [4.8750, 4.8750] | 0.758947 | 0.7559  [0.7558, 0.7561] | 85.68009 | 84.9683  [84.9700, 84.9700] | 17.77202 | 17.4297  [17.4300, 17.4300] |
| 25.00 | 4.814567 | 4.8751  [4.8750, 4.8750] | 0.807417 | 0.8048  [0.8047, 0.8049] | 85.76028 | 84.9748  [84.9700, 84.9800] | 17.81267 | 17.4303  [17.4300, 17.4300] |
