# Supplementary material for: A Discrete Event Simulation Model for Evaluating the Performances of an M/G/C/C State Dependent Queuing System
Source: PLoS One. 2013 Apr 1;8(4):e58402. doi: 10.1371/journal.pone.0058402 (PMC3613361; doi:10.1371/journal.pone.0058402)
Supplement: Appendix S6 — Comparison between Analytic and Simulation for Corridor 11. (DOCX) [file pone.0058402.s006.docx]

**Appendix S6** Comparison between Analytic and Simulation for Corridor 11

| λ | Ө | | p(c) | | L | | W | |
| --- | --- | --- | --- | --- | --- | --- | --- | --- |
|  | Analytic | Simulation | Analytic | Simulation | Analytic | Simulation | Analytic | Simulation |
| 1.00 | 1.0000 | 1.0000  [0.9977, 1.0020] | 0.0000 | 0.0000  [0.0000, 0.0000] | 1.5926 | 1.5929  [1.5890, 1.5970] | 1.5926 | 1.5928  [1.5920, 1.5930] |
| 1.50 | 1.5000 | 1.5016  [1.4980, 1.5050] | 0.0000 | 0.0000  [0.0000, 0.0000] | 2.4555 | 2.4585  [2.4520, 2.4640] | 1.6370 | 1.6373  [1.6370, 1.6380] |
| 2.00 | 2.0000 | 1.9987  [1.9960, 2.0020] | 0.0000 | 0.0000  [0.0000, 0.0000] | 3.3730 | 3.3711  [3.3650, 3.3770] | 1.6865 | 1.6867  [1.6860, 1.6870] |
| 2.50 | 2.5000 | 2.5023  [2.4980, 2.5070] | 0.0000 | 0.0000  [0.0000, 0.0000] | 4.3547 | 4.3599  [4.3500, 4.3700] | 1.7419 | 1.7423  [1.7420, 1.7430] |
| 3.00 | 3.0000 | 3.0012  [2.9970, 3.0050] | 0.0000 | 0.0000  [0.0000, 0.0000] | 5.4136 | 5.4165  [5.4080, 5.4250] | 1.8045 | 1.8048  [1.8040, 1.8050] |
| 3.50 | 3.5000 | 3.5032  [3.4980, 3.5080] | 0.0000 | 0.0000  [0.0000, 0.0000] | 6.5665 | 6.5737  [6.5610, 6.5860] | 1.8761 | 1.8765  [1.8750, 1.8780] |
| 4.00 | 4.0000 | 3.9975  [3.9910, 4.0040] | 0.0000 | 0.0000  [0.0000, 0.0000] | 7.8377 | 7.8289  [7.8110, 7.8470] | 1.9594 | 1.9584  [1.9570, 1.9600] |
| 4.50 | 4.5000 | 4.4940  [4.4880, 4.5000] | 0.0000 | 0.0000  [0.0000, 0.0000] | 9.2637 | 9.2443  [9.2250, 9.2630] | 2.0586 | 2.0570  [2.0560, 2.0580] |
| 5.00 | 5.0000 | 5.0010  [4.9940, 5.0080] | 0.0000 | 0.0000  [0.0000, 0.0000] | 10.9050 | 10.9075  [10.8800, 10.9300] | 2.1810 | 2.1810  [2.1790, 2.1830] |
| 5.50 | 5.4998 | 5.4697  [5.4160, 5.5230] | 0.0000 | 0.0000  [-0.0038, 0.0152] | 12.8948 | 14.5861  [11.7300, 17.4400] | 2.3446 | 2.7084  [2.0840, 3.3320] |
| 6.00 | 5.9766 | 4.9306  [4.8170, 5.0440] | 0.0039 | 0.1879  [0.1586, 0.1967] | 16.3810 | 51.7996  [47.9200, 55.6800] | 2.7409 | 10.6587  [9.6930, 11.6200] |
| 6.10 | 6.0457 | 4.8315  [4.7240, 4.9390] | 0.0089 | 0.1959  [0.1899, 0.2256] | 18.0245 | 56.0212  [52.5600, 59.4800] | 2.9814 | 11.7323  [10.8400, 12.6200] |
| 6.20 | 6.0801 | 4.7769  [4.7000, 4.8530] | 0.0193 | 0.2195  [0.2168, 0.2414] | 20.6604 | 58.3315  [56.1100, 60.5600] | 3.3980 | 12.2813  [11.6500, 12.9100] |
| 6.30 | 6.0520 | 4.6312  [4.5950, 4.6680] | 0.0394 | 0.2707  [0.2588, 0.2705] | 24.8567 | 62.8208  [61.8100, 63.8300] | 4.1072 | 13.5820  [13.2600, 13.9000] |
| 6.40 | 5.9327 | 4.5943  [4.5730, 4.6160] | 0.0730 | 0.2816  [0.2778, 0.2849] | 31.0118 | 63.9577  [63.4000, 64.5200] | 5.2272 | 13.9270  [13.7400, 14.1100] |
| 6.50 | 5.7179 | 4.5673  [4.5500, 4.5850] | 0.1203 | 0.2976  [0.2940, 0.2998] | 38.7446 | 64.7160  [64.2800, 65.1500] | 6.7760 | 14.1735  [14.0300, 14.3200] |
| 6.60 | 5.4501 | 4.5403  [4.5290, 4.5510] | 0.1742 | 0.3131  [0.3102, 0.3140] | 46.6673 | 65.4268  [65.1800, 65.6800] | 8.5626 | 14.4119  [14.3200, 14.5000] |
| 6.70 | 5.1951 | 4.5452  [4.5370, 4.5530] | 0.2246 | 0.3202  [0.3199, 0.3224] | 53.2602 | 65.3270  [65.1500, 65.5000] | 10.2519 | 14.3735  [14.3100, 14.4400] |
| 6.80 | 4.9953 | 4.5332  [4.5270, 4.5390] | 0.2654 | 0.3334  [0.3319, 0.3343] | 57.8975 | 65.6129  [65.4800, 65.7400] | 11.5905 | 14.4742  [14.4300, 14.5200] |
| 6.90 | 4.8562 | 4.5324  [4.5260, 4.5390] | 0.2962 | 0.3424  [0.3413, 0.3438] | 60.8247 | 65.6428  [65.5200, 65.7600] | 12.5252 | 14.4834  [14.4400, 14.5300] |
| 7.00 | 4.7647 | 4.5274  [4.5240, 4.5310] | 0.3193 | 0.3519  [0.3515, 0.3534] | 62.5775 | 65.7551  [65.6800, 65.8300] | 13.1336 | 14.5241  [14.5000, 14.5500] |
| 8.00 | 4.5628 | 4.5180  [4.5180, 4.5180] | 0.4297 | 0.4346  [0.4343, 0.4353] | 65.5398 | 65.9382  [65.9300, 65.9400] | 14.3640 | 14.5947  [14.5900, 14.6000] |
| 9.00 | 4.5244 | 4.5172  [4.5170, 4.5170] | 0.4973 | 0.4985  [0.4976, 0.4984] | 65.9354 | 65.9521  [65.9500, 65.9600] | 14.5731 | 14.6004  [14.6000, 14.6000] |
| 10.00 | 4.5035 | 4.5166  [4.5160, 4.5170] | 0.5496 | 0.5478  [0.5476, 0.5483] | 66.1507 | 65.9614  [65.9600, 65.9600] | 14.6886 | 14.6041  [14.6000, 14.6000] |
| 15.00 | 4.4630 | 4.5158  [4.5160, 4.5160] | 0.7025 | 0.6987  [0.6986, 0.6990] | 66.5707 | 65.9661  [65.9700, 65.9700] | 14.9161 | 14.6079  [14.6100, 14.6100] |
| 20.00 | 4.4495 | 4.5156  [4.5160, 4.5160] | 0.7775 | 0.7739  [0.7739, 0.7741] | 66.7115 | 65.9668  [65.9700, 65.9700] | 14.9931 | 14.6087  [14.6100, 14.6100] |
| 25.00 | 4.4427 | 4.5155  [4.5150, 4.5160] | 0.8223 | 0.8193  [0.8193, 0.8195] | 66.7826 | 65.9671  [65.9700, 65.9700] | 15.0321 | 14.6090  [14.6100, 14.6100] |
